# Hole doping effect of MoS_2_ via electron capture of He^+^ ion irradiation

**DOI:** 10.1038/s41598-021-02932-6

**Published:** 2021-12-08

**Authors:** Sang Wook Han, Won Seok Yun, Hyesun Kim, Yanghee Kim, D.-H. Kim, Chang Won Ahn, Sunmin Ryu

**Affiliations:** 1 Department of Physics and Energy Harvest Storage Research Center, University of Ulsan, Ulsan, 44610 Republic of Korea; 2 Convergence Research Institute, DGIST, Daegu, 42988 Republic of Korea; 3 Department of Chemistry, Pohang University of Science and Technology (POSTECH), Pohang, Gyeongbuk 37673 Republic of Korea; 4 Pohang Accelerator Laboratory, Beamline Research Division, Pohang, Gyeongbuk 37673 Republic of Korea

**Keywords:** Two-dimensional materials, Two-dimensional materials, Surfaces, interfaces and thin films

## Abstract

Beyond the general purpose of noble gas ion sputtering, which is to achieve functional defect engineering of two-dimensional (2D) materials, we herein report another positive effect of low-energy (100 eV) He^+^ ion irradiation: converting n-type MoS_2_ to p-type by electron capture through the migration of the topmost S atoms. The electron capture ability via He^+^ ion irradiation is valid for supported bilayer MoS_2_; however, it is limited at supported monolayer MoS_2_ because the charges on the underlying substrates transfer into the monolayer under the current condition for He^+^ ion irradiation. Our technique provides a stable and universal method for converting n-type 2D transition metal dichalcogenides (TMDs) into p-type semiconductors in a controlled fashion using low-energy He^+^ ion irradiation.

## Introduction

After graphene was initially actively studied^1,2^, an atomically thin monolayer MoS_2_ has since attracted intensive attention due to its novel features, such as thickness-dependent indirect-to-direct bandgap transition and lattice vibration^3–5^. In particular, the successful realization of field-effect transistors composed of monolayer MoS_2_ has facilitated the development of high-performance flexible electronic and optoelectronic devices^6–8^. However, in addition to accumulated excess electrons on the MoS_2_ surface^9^, strong Fermi level pinning and high contact resistance between the metal electrode and underlying few- and monolayer MoS_2_ predominantly induce n-type unipolar conductivity^10–13^. Various approaches to converting n-type TMDs to p-type have been proposed and employed, such as substitution, intercalation, charge transfer, and electrostatic doping^14,15^. Doping technology plays an essential role in the semiconductor electronics industry. Ion implantation and thermal diffusions are traditional doping methods that are used to precisely control the carrier type and concentration of three-dimensional semiconductors. However, the recent emergence of 2D materials has necessitated the development of an efficient and facile doping methodology that avoids structural damage problems due to their low dimensionality. The use of these materials also requires overcoming unavoidable contamination, a complicated transfer process, and inevitable defects to retain the novel functionalities of 2D TMDs^16,17^.

To search for a more practical, stable, and simple doping method, we revisited the literature regarding Ar^+^ or He^+^ ion sputtering to convert the n-type MoS_2_ surface into a p-type semiconductor^18^. Generally, noble gas ion sputtering can tailor various defect types on the basal plane of TMDs depending on the incidence angle, ion mass, and ion energy^19,20^. The creation of mono-sulfur vacancy defects on the inert basal plane of the monolayer MoS_2_ by Ar^+^ ion sputtering has been shown to enable maximized activation for the hydrogen evolution reaction^21^. The He^+^ ion can also etch and pattern TMDs and further induce the insulator-to-metal transition^22–27^. Notably, these employed He ion energies are much larger than the minimum energies required to sputter S atoms (17.4 eV) and Mo atoms (130.2 eV) based on the calculated displacement thresholds for S (~ 6.9 eV) and Mo (~ 20 eV) in MoS_2_^26,28^. In this study, unlike the approach employing defect engineering of 2D TMDs using high-energy noble gas ion sputtering, we report another positive effect of low-energy (100 eV) He^+^ ion irradiation that can be highly tuned to capture accumulated excess electrons on the n-type MoS_2_ surfaces. The photoemission and work function (WF) measurements have shown p-doping shifts of the Fermi energy (E_F_) from the bulk to bilayer MoS_2_ surfaces by He^+^ ion irradiation. By contrast, the monolayer MoS_2_ surfaces have induced further n-doping shifts due to the over-attracted charges from the underlying substrates as a result of the He^+^ ion irradiation. Finally, Raman and photoluminescence (PL) measurements and theoretical first-principles calculations have demonstrated that the migration of the topmost S atoms by low-energy He^+^ ion irradiation increases the bandgap size of the MoS_2_ surface.

## Experimental and computational details

The synchrotron-based photoemission spectroscopy measurements and He^+^ ion sputtering were performed at the 10D beamline of the Pohang Light Source (PLS) in South Korea. All photoemission data were obtained in the ultra-high vacuum chamber of ~ 3 × 10^−10^ Torr using an R4000 analyzer (VG-Scienta) at room temperature; they were all normalized to the incident photon flux. The binding energies were derived by measuring the E_F_ of the gold films. The energy and angle resolutions of the angle-resolved photoemission spectroscopy (ARPES) apparatus for the data obtained at the photon energy of 56 eV were 100 meV and 0.05°, respectively. In the curve fitting of the core-level spectra^29^, Shirley background subtraction and Doniach-Šunjić functions were used to derive the natural (Lorentzian) and Gaussian line widths that respectively represent the core–hole lifetime and the instrumental energy resolution^30^. For simplicity, after being derived from the Au 4*f*. core-level spectra, the Gaussian widths were fixed at 0.455 eV for Mo 3*d* (*hν* = 300 eV) and 0.355 eV for S 2*p* (*hν* = 222 eV) core-level spectra. The values of the spin–orbit coupling and the branching ratios for S 2*p* [I(2*p*_3/2_)/I(2*p*_1/2_)] and Mo 3*d* [I(3*d*_5/2_)/I(3*d*_3/2_)] core-level spectra were (1.18 and 3.13) eV and (0.5 and 0.67), respectively.

For the He^+^ ion irradiation, the samples were transferred into the preparation chamber while maintaining a base pressure of ~ 5 × 10^−10^ Torr using automated sample transfers. Then, the He gas (99.9999%) was filled into the preparation chamber by 10^−6^ Torr through the ion sputter gun (SPECS IQE 11-A). The ion energy and irradiation time were varied at a 45° angle of incidence.

Single-crystalline 2H-MoS_2_ crystals (SPI, natural molybdenite) were irradiated by He^+^ ion after being in-situ cleaved. However, the centimeter-scale MoS_2_ bi- and monolayers CVD-grown on the SiO_2_/Si and Al_2_O_3_ substrates (purchased from 2D semiconductors) and the mechanically exfoliated MoS_2_ flakes were irradiated by He^+^ ion after exposure to ambient air. Notably, to compensate for the charging effect during the photoemission measurements, the surfaces of the large-scale CVD-grown samples on the insulating substrates were contacted by Au wires attached to the sample holder.

The work functions were measured in ambient and dry nitrogen using a Kelvin probe system (APS01, KP Technology) with an excitation range from 3.3 eV to 6.8 eV that was calibrated from the contact potential difference between the gold surface and tip.

Using the micromechanical exfoliation method^5^, few- and monolayer MoS_2_ samples were deposited onto Si substrates with a 285-nm-thick SiO_2_ layer by exfoliating bulk 2H-MoS_2_ crystals. The film’s thickness was characterized through Raman spectra obtained with an Ar ion laser at 514 nm; the same laser also generated the PL signals. In both measurements, the average power was maintained below 0.2 mW to avoid irreversible photoinduced changes. Lorentzian functions were used in curve-fitting PL and Raman spectra.

The calculation was performed using density functional theory with the projector augmented wave (PAW) method^31,32^, as implemented in the Vienna *ab-initio* simulation package (VASP) code^33^. Plane waves with an energy cut-off of 500 eV were used to expand the Kohn–Sham orbitals. In addition, exchange and correlation interactions between electrons were described with the generalized gradient approximation (GGA)^34^. Integration over the Brillouin zone was carried out using a 4 × 4 × 1 k-points mesh while employing a vacuum space of more than 20 Å for the 4 × 4 supercell systems. Further, all geometries were optimized using the conjugate gradient method with van der Waals correction (optB88-vdW functional)^35^.

## Results and discussion

**Figure 1:**
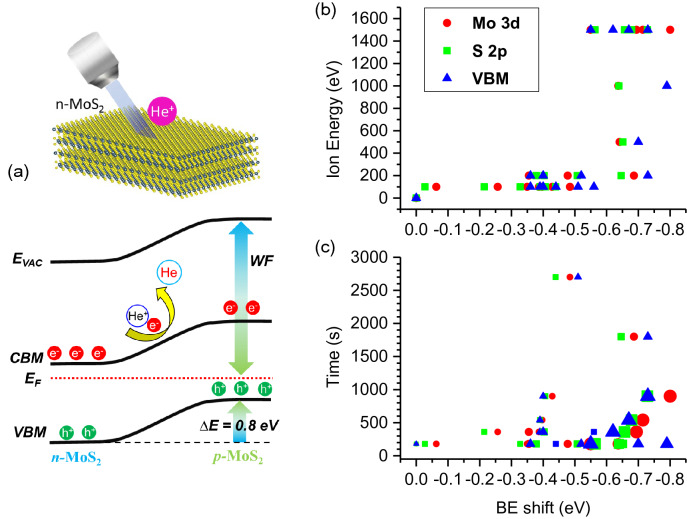
(**a**) Diagram depicting the conversion of n-type MoS_2_ to p-type via the downward shifts of Fermi energy (E_F_) level and the increase in the work function (WF) from the electron capture of the He^+^ ion irradiation on the MoS_2_ surface (inset). (**b**,**c**) The negative binding energy shifts of Mo 3*d*, S 2*p*, and valence band photoemission spectra depend on the ion energy (**b**) and irradiation time (**c**) of He^+^ ion irradiation. (**c**) Symbol size indicates the increase of the ion energy from 0.1 eV (left) to 1.5 keV (right).

As demonstrated in Fig. 1a, in n-type MoS_2_ surfaces, He^+^ ion irradiation using a commercial sputter ion gun converts p-type semiconductors by shifting the E_F_ downward and increasing the WF. Figure 1b,c show that, as the He^+^ ion beam energy or irradiation time increased, all the binding energies of Mo 3*d* and S 2*p* core-level spectra, as well as the valence band maxima (VBMs) obtained from the in-situ cleaved surfaces of MoS_2_ single crystals, negatively shifted (Supplementary Figs. S1–S4). It is worth noting that the He gas exposure did not change the photoemission spectra of MoS_2_ when the sputter gun was turned off (Supplementary Fig. S1). The maximum binding energy shift was − 0.8 eV by the relatively high-energy (1.5 keV) He^+^ ion irradiation with a longer time (900 s) (Supplementary Fig. S2). The lowest stable He^+^ ion energy was 100 eV using a commercial sputter ion gun (Supplementary Figs. S1 and S4). Consequently, we focus on all the experimental results of this low-energy (100 eV) He^+^ ion irradiation condition.

**Figure 2:**
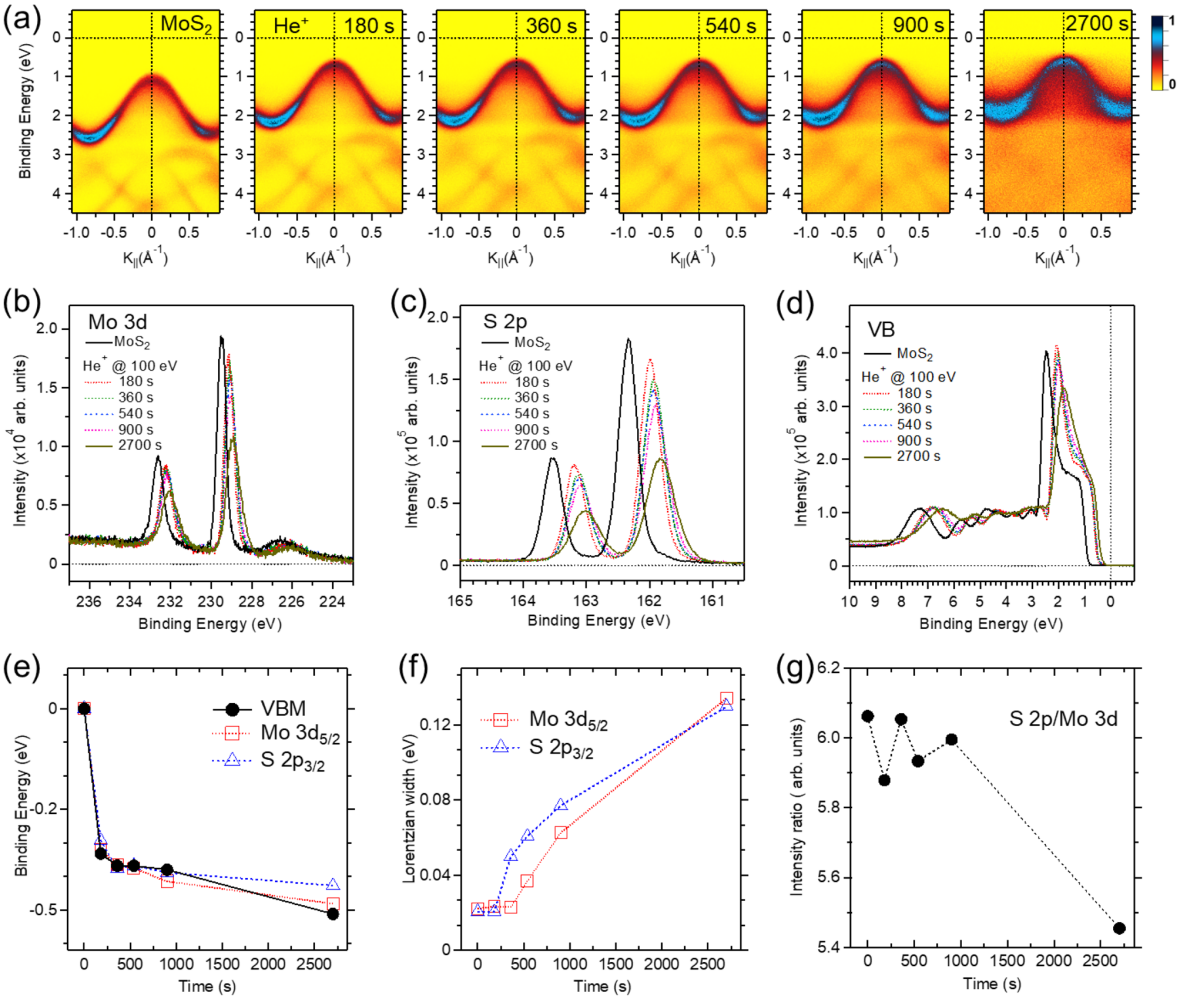
(**a**) ARPES intensity maps along the $\overline{\mathrm{M\Gamma K} }$ symmetry lines were taken at the photon energy of *hν* = 56 eV. The overall band structure of the freshly cleaved n-type surface of the MoS_2_ single crystal moved upward from left to right as a function of the He^+^ ion irradiation time at the fixed low ion energy of 100 eV. (**b**–**d**) Comparison of Mo 3*d*, S 2*p*, and valence-band photoemission spectra taken at the respective photon energies of *hν* = 300 eV, 222 eV, and 56 eV. (**e**) Binding energy shifts of the prominent Mo 3*d*_5/2_ and S 2*p*_3/2_ peaks and the VBM. The binding energies of the cleaved MoS_2_ surface are 229.50 eV, 162.34 eV, and 0.87 eV, respectively. (**f**) Comparison of Lorentzian linewidth of Mo 3*d* and S 2*p* core-level spectra. (**g**) The intensity ratio of S 2*p* to Mo 3*d* core-level spectra.

Figure 2a exhibits a rigid energy shift of the overall band structure of the in-situ cleaved surface of a MoS_2_ single crystal toward the E_F_ with increased He^+^ ion irradiation time. The band dispersion of the He^+^ ion irradiated MoS_2_ surface became blurred at the final stage. The comparison of Mo 3*d* and S 2*p* core-level spectra and valence band spectra indicates the broadening linewidth and decreasing intensity caused by He^+^ ion irradiation (Fig. 2b–d).

According to the curve-fitting results, all binding energies of the prominent peaks of Mo 3*d*_5/2_ and S 2*p*_3/2_, as well as the VBMs, relatively shifted up to − 0.5 eV at the final stage (Fig. 2e). The other 2D materials of MoSe_2_ and SnS_2_ also showed a rigid energy shift due to He^+^ ion irradiation (Supplementary Figs. S5 and S6). Concurrently, the Lorentzian linewidths of the Mo 3*d* and S 2*p* core-level spectra were systematically broadened by the irradiation of He^+^ ion (Fig. 2f). The Lorentzian width of the S 2*p* peak broadens before the Mo 3*d* peak because the displacement threshold of S is much lower than that for Mo^26,28^. Considering that we have limited the Gaussian width contributing to the experimental resolution and other physical effects for the simplicity of the curve-fitting, the Lorentzian broadening upon He^+^ ion irradiation suggests crystallinity disorder and the production of S or Mo vacancy defect. The intensity ratio of the S 2*p* to Mo 3*d* peaks, which is directly related to the stoichiometry of the MoS_2_ surface, can be used to distinguish between these two statuses (Fig. 2g). The oscillating intensity ratio is related to the random migration of S or Mo atoms. The significant reduction at the final stage implies that He^+^ ion irradiation preferentially sputtered the topmost S atoms^36^.

**Figure 3:**
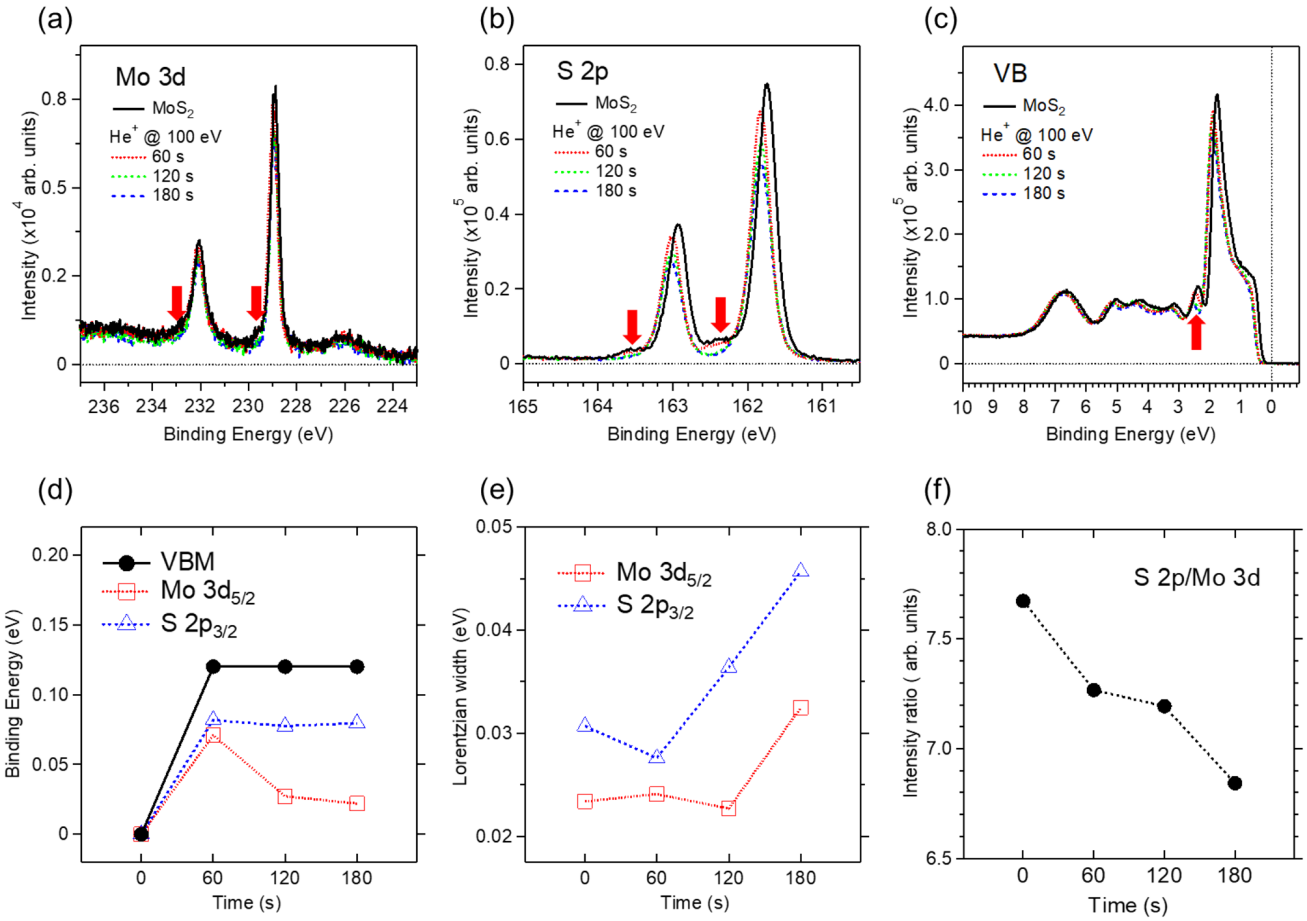
(**a**–**c**) Comparison of Mo 3*d*, S 2*p*, and valence-band photoemission spectra obtained by in-situ He^+^ ion irradiation on the freshly cleaved surface of the MoS_2_ single crystal. These spectra were taken at the photon energies of *hν* = 300 eV, 222 eV, and 56 eV, respectively. The red arrows beside the prominent p-type peaks indicate the n-type features (see the context for details). (**d**) The binding energy shifts of the prominent Mo 3*d*_5/2_ and S 2*p*_3/2_ peaks and the VBM. The binding energies of the cleaved MoS_2_ surface are 228.92 eV, 161.74 eV, and 0.30 eV, respectively. (**f**) Comparison of Lorentzian linewidth of Mo 3*d* and S 2*p* core-level spectra. (**g**) The intensity ratio of the S 2*p* to Mo 3*d* core-level spectra.

On the other hand, we occasionally found a p-type MoS_2_ surface from several investigated single crystals (Fig. 3). Compared to the n-type MoS_2_ surfaces (Fig. 2 and Supplementary Figs. S1–S4), the photoemission spectra of the p-type MoS_2_ surface all appeared at binding energies that were lower by − 0.6 eV (Fig. 3a–c)^37–39^. Further, the He^+^ ion irradiation on the p-type MoS_2_ surface reversely shifted the photoemission spectra toward the high binding energy side (Fig. 3d). Concurrently, the weak peaks, which are indicated by arrows in the figure (Fig. 3a–c), either were weakened or disappeared after the He^+^ ion irradiation. In another sample (Supplementary Fig. S4), the splitting of prominent peaks into high (n-type) and low (p-type) binding energy components by He^+^ ion irradiation supports that the weak peaks correlate to n-type features^12^.

The faster Lorentzian broadening for S 2*p* compared to Mo 3*d*, along with the decreasing intensity ratio of S 2*p* to Mo 3*d* peaks, indicates the continuous sputtering of S atoms (Fig. 3e,f). Nevertheless, the intensity ratios of the initial and final values (~ 7) are more significant than those (~ 6) of most n-type MoS_2_ surfaces. According to the experimentally observed modulating carrier type depending on the stoichiometry^12^, the n- and p-type MoS_2_ surfaces are relatively sulfur-deficient and sulfur-rich, respectively. In addition, our calculations intuitively support the carrier type dependence on stoichiometry (Supplementary Fig. S7). The most frequently occurring mono-sulfur vacancy defect induces significant in-gap defect states of Mo *d* orbitals below the conduction band minimum (CBM) of the defect-free MoS_2_ monolayer^40–44^. By contrast, the S atom’s adsorption above the S atom of the monolayer MoS_2_ enhances the S *p* orbitals at the VBM in a manner comparable to the Mo *d* orbitals. The additional neighboring S atom above the Mo atom of the monolayer MoS_2_ causes in-gap defect states of S *p* orbitals below the CBM. The removal and addition of the S atom to the defect-free MoS_2_ monolayer correspond to the contrasting ionizing dopants between the n-type (Mo^4+^) and p-type (S^2−^).

**Figure 4:**
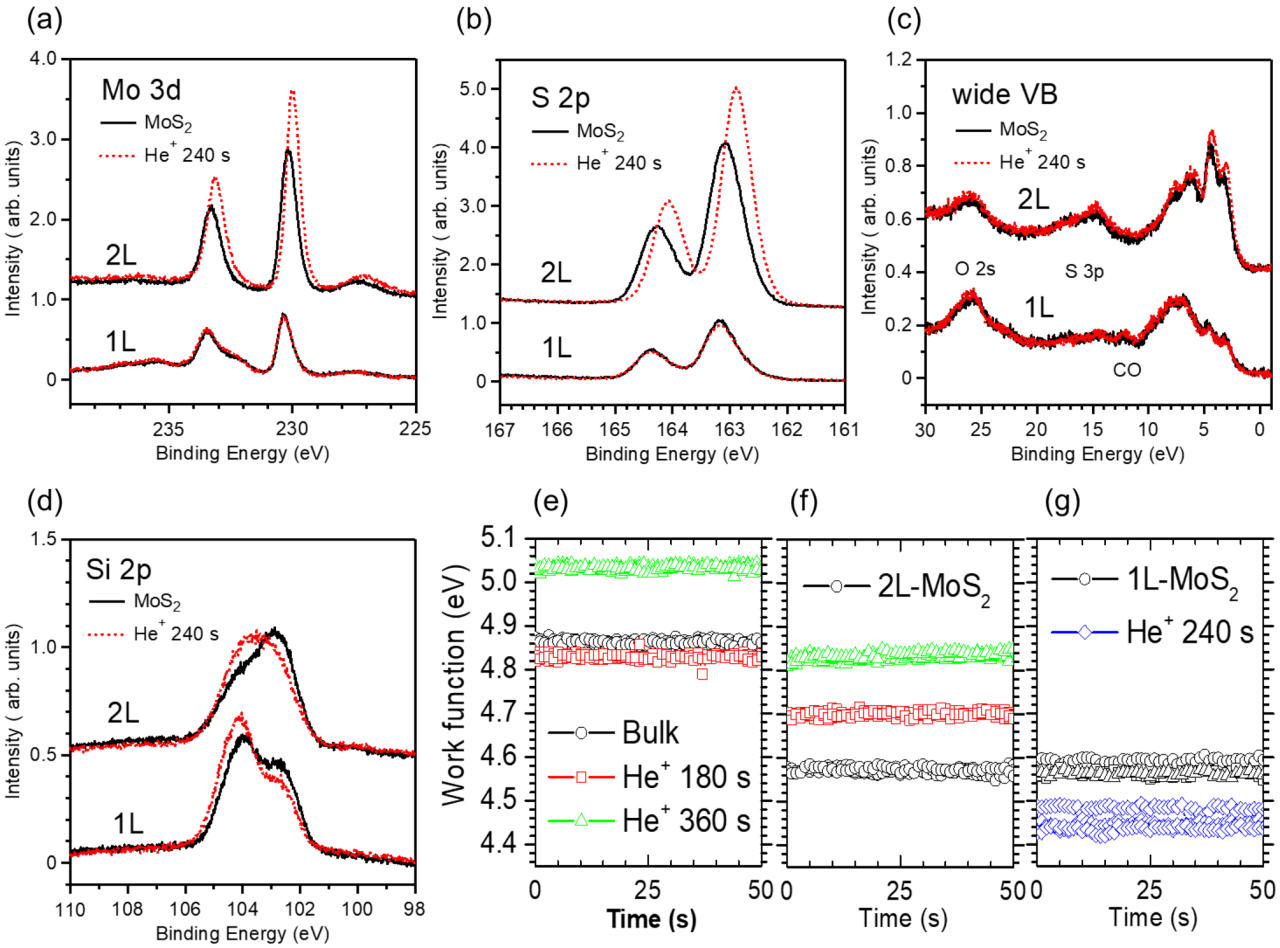
(**a**–**d**) Comparison of photoemission spectra between the CVD-grown 1L- and 2L-MoS_2_ samples before and after low-energy (100 eV) He^+^ ion irradiation. The photoemission data were taken at the photon energy of *hν* = 360 eV. (**e**–**g**) The work functions were measured at three random positions for each sample using an ambient Kelvin probe system.

We also investigated the inevitably air-exposed MoS_2_ samples to elucidate the influences of the He^+^ ion irradiation on the WF values, structural properties, and bandgap changes. Figure 4a–c compare the photoemission spectra between the large-scale bilayer and monolayer MoS_2_ samples. They were CVD-grown on the SiO_2_/Si substrates but exposed to ambient air. The He^+^ ion irradiation enhanced and shifted Mo 3*d*, S 2*p*, and the valence-band photoemission spectra of the bilayer MoS_2_ toward lower binding energies, but it rarely changed those of the monolayer MoS_2_. By contrast, the Si 2*p* core-level spectra of both substrates slightly shifted toward higher binding energies after the He^+^ ion irradiation (Fig. 4d). The Si 2*p* core-level spectra constitute two peaks at the high and low binding energies, respectively corresponding to the SiO_2_ (Si^4+^) substrate and the suboxide of SiO_*x*_ (Si^3+^)^45,46^. The smaller and larger Si^4+^ peaks than the Si^3+^ peaks in the bilayer and monolayer MoS_2_ samples increased after the He^+^ ion irradiation. The SiO_2_/Si substrates are supposed to act as charge reservoirs and serve as n-type doping into the monolayer MoS_2_^47^. This then appears to result in a lack of MoS_2_-related photoemission spectra shifts in the monolayer under the He^+^ ion irradiation’s current condition. Alternatively, this may also be caused by the severely contaminated surface of the monolayer MoS_2_. The oxide peaks, which are indicative of the molybdenum oxidation state of Mo^6+^ above the Mo 3*d*^3/2^ spectra (Fig. 4a), O 2*s* core-level spectra, and the CO features (Fig. 4c), were much more overwhelming in the monolayer than in the bilayer; this will be discussed in detail in a later section.

To confirm the modulating carrier type that is expected from the photoemission measurements before and after He^+^ ion irradiation, we conducted WF measurements and prepared additional MoS_2_ samples. The WF is the difference between the local vacuum energy and E_F_ (Fig. 1a). The WF values of the bulk (4.86 eV), bilayer (4.57 eV), and monolayer (4.59 eV) (Fig. 4e–g) were comparable to those reported in prior studies^48–52^. These WF values of the bulk and bilayer MoS_2_ samples increased after the He^+^ ion irradiation; however, that of the monolayer MoS_2_ sample decreased. These contrasting results support the photoemission data indicating the p-type doping in the bulk and bilayer MoS_2_ samples and further n-type doping in the monolayer MoS_2_ sample. More interestingly, these air-exposed samples provide a method that can be applied in practice to convert the n-type MoS_2_ surfaces into p-type under the current condition of the He^+^ ion irradiation, even though it is valid over the bilayer thickness.

Finally, the Raman and PL spectroscopy measurements elucidate whether or not the n-type to p-type (or further n-type) conversion by the He^+^ ion irradiation observed from the MoS_2_-related photoemission spectra and WF values is related to the creation of structural defects together with the bandgap change. There were no Raman or PL spectra shifts between the air-exposed and He^+^ ion-irradiated samples (Supplementary Figs. S8–S11). However, compared to the changes in Raman intensity for most bulk MoS_2_ crystals and CVD-grown bilayer and monolayer samples by the He^+^ ion irradiation, the PL intensities were significantly reduced. Specifically, the He^+^ ion irradiation further decreased the PL intensities of the CVD-grown bilayer on the Al_2_O_3_ substrate compared to those of the CVD-grown bilayer on the SiO_2_ substrate. Remarkably, the He^+^ ion irradiation exhibited red-shifts in both the Raman and PL data of the CVD-grown monolayer MoS_2_ sample on the SiO_2_ substrate compared to those of the Al_2_O_3_ substrate (Supplementary Fig. S11). The corresponding optical microscope images indicate that the CVD-grown MoS_2_ monolayers on the SiO_2_ substrate are prone to be degraded from extensive cracking due to the oxidation along the grain boundaries^53^. This may be a possible reason for the lack of change in the photoemission data of the monolayer MoS_2_ by the He^+^ ion irradiation. To circumvent this problem, we prepared the mechanically exfoliated MoS_2_ monolayers on the SiO_2_ substrates.

**Figure 5:**
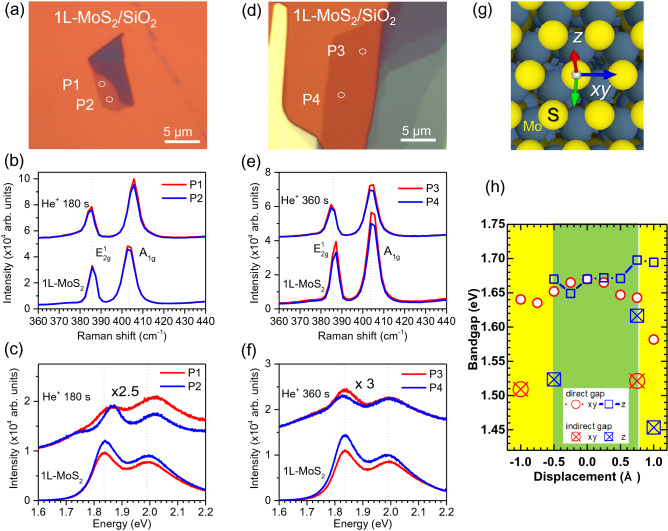
(**a**,**d**) Optical microscope images of the mechanically exfoliated MoS_2_ flakes on the SiO_2_/Si substrates. Comparison of Raman (**b**,**e**) and PL (**c**,**f**) spectra of 1L-MoS_2_ samples before and after He^+^ ion irradiation for 180 s (**b**,**c**) and 360 s (**e**,**f**). (**g**) Schematic of available migrations for the S atoms before sputtering with He^+^ ion irradiation. (**h**) Displacement dependence of the S atom on the direct bandgap energies of 1L-MoS_2_.

Figure 5 compares the Raman and PL data obtained at the same positions before and after the He^+^ ion irradiation for 180 s (Fig. 5a–c) and 360 s (Fig. 5d–f). As the He^+^ ion irradiation time increased, the PL intensities decreased significantly compared to the Raman intensities. Considering the enhanced PL intensity of the monolayer MoS_2_ by p-type doping^54^, the electron doping into the monolayer MoS_2_ attracted from the underlying SiO_2_ substrate by the He^+^ ion irradiation could reduce the PL intensities. For the He^+^ ion irradiation time of 180 s, the difference between the E^1^_2g_ and A_1g_ Raman peaks increased by the former and latter’s red- and blue shifts (Fig. 5b). The red- and blue Raman shifts are attributed to the tensile and compressive strains by decreasing and increasing the force constant in MoS_2_, respectively^55^. Besides, the increased separation between two Raman peaks is supposed to have resulted from vacancy defects^55^. Accordingly, the direct A and B exciton peaks’ energies of the PL spectra increased, thus forming the shoulders below the A peak (Fig. 5c). For the He^+^ ion irradiation time of 360 s, the differences between the E^1^_2g_ and A_1g_ Raman peaks increased less due to slight red-shifts of both peaks (Fig. 5e). Thus, the A and B exciton peaks’ energies did not change while retaining the shoulders below the A peak (Fig. 5f). PL is highly dependent on doping^56^. These trion-like features can be formed by injected carriers from the SiO_2_ substrates into the monolayer MoS_2_ or in-gap defect states below the CBM by removing and adding the S atom through He^+^ ion irradiation^56^ (Supplementary Fig. S7). Notably, the intensity ratio of the E^1^_2g_ peak to the A_1g_ peak and their Lorenzian widths all increased for the He^+^ ion irradiation of 180 s but decreased for 360 s. The optical microscope image indicates damaged areas near these positions after 360 s of He^+^ ion irradiation (Supplementary Fig. S12). The Raman intensities further decreased, but the difference between the E^1^_2g_ and A_1g_ peaks increased by their respective red- and blue shifts, as shown in the He^+^ ion irradiated sample for 180 s (Fig. 5b). However, the PL signals disappeared. This may be due to the topmost layer’s desulfurization^36^, which can lead to the metallic property as a result of the exposed Mo atoms, as shown in bulk MoS_2_ (Supplementary Fig. S8). The He^+^ ion irradiation time for 180 s seems to be the critical parameter for generating the S vacancy defects. The low concentration of S vacancy defects increases the direct bandgap of the defect-free MoS_2_, but it also leads to an increase in the indirect bandgap by inducing in-gap defect states^44^. Another possibility, based on the increasing intensity ratio of S 2*p* to Mo 3*d* peaks, is the migration of the topmost S atoms before sputtering by He^+^ ion irradiation (Supplementary Figs. S1–S4). When the S atom moves along the horizontal direction (Fig. 5g), the direct bandgap size of the monolayer MoS_2_ decreases (Fig. 5h). However, the bandgap size increases when moving along the positive vertical direction. The direct bandgap becomes indirect if the displacement is outside the ranges of − 0.5 Å and 0.75 Å. The size of the indirect bandgap reduces with the enhanced VBM/CBM or in-gap defect states consisting of Mo *d* orbitals (Supplementary Fig. S13). Therefore, converting n-type MoS_2_ to p-type by electron capture under the current low-energy (100 eV) He^+^ ion irradiation is supposed to be accompanied by the migration of the topmost S atoms or its minimal sputtering. We note that it is possible to lower the kinetic energies of He^+^ ions down to ~ 15 eV by using a commercial sputter ion gun equipped with a Wien-filter for mass/charge separation^57^. Further research needs to study the possibility of converting n-type supported MoS_2_ monolayer into p-type without deteriorating its intrinsic functionality by using much lower energy of He^+^ ion irradiation or using a capping layer like the overlayer graphene under the current He^+^ ion irradiation^29^.

## Summary

In conclusion, the photoemission and WF measurements have shown p-doping shifts of the E_F_ from the bulk to bilayer MoS_2_ surfaces via the electron capture of the low-energy (100 eV) He^+^ ion irradiation. Meanwhile, the electron capture ability of the He^+^ ion irradiation is retained up to the MoS_2_ bilayer thickness, but it is restricted at the MoS_2_ monolayer because it attracts electrons from underlying substrates. Raman and photoluminescence measurements and theoretical first-principles calculations have revealed that the p-doping effects by the electron capture of the low-energy He^+^ ion irradiation are attributed to the increased bandgap size of the MoS_2_ surface through the migration of the topmost S atoms. Moreover, the p-type capacitance–voltage curves of the thicker MoS_2_ metal–oxide–semiconductor capacitor (Supplementary Fig. S14) concretize that the He^+^ ion irradiation could be a stable and universal method for achieving the ultimate performances of the MoS_2_-based p-type FET device^58^.

## Supplementary Information

Supplementary Information.
