# Supplementary material for: Hole doping effect of MoS2 via electron capture of He+ ion irradiation
Source: Sci Rep. 2021 Dec 8;11:23590. doi: 10.1038/s41598-021-02932-6 (PMC8654839; doi:10.1038/s41598-021-02932-6)
Supplement: Supplementary file 1 — Supplementary Information. [file 41598_2021_2932_MOESM1_ESM.pdf]

Supplementary Information:

Hole doping effect of MoS_2 via electron capture of He^+ ion irradiation

Sang Wook Han,[1,5,*] Won Seok Yun,[2,5] Hyesun Kim,[3] Yanghee Kim,[3] D.-H. Kim,[4] Chang Won Ahn,[1] and S. Ryu[3]

[1]Department of Physics and Energy Harvest Storage Research Center, University of Ulsan, Ulsan 44610, Republic of Korea

[2]Convergence Research Institute, DGIST, Daegu 42988, Republic of Korea

[3]Department of Chemistry, Pohang University of Science and Technology (POSTECH), Pohang, Gyeongbuk 37673, Republic of Korea

[4]Beamline Research Division, Pohang Accelerator Laboratory, Pohang, Gyeongbuk 37673, Republic of Korea

[5]These authors contributed equally: Sang Wook Han and Won Seok Yun.

[*]Corresponding author: swhan72@ulsan.ac.kr

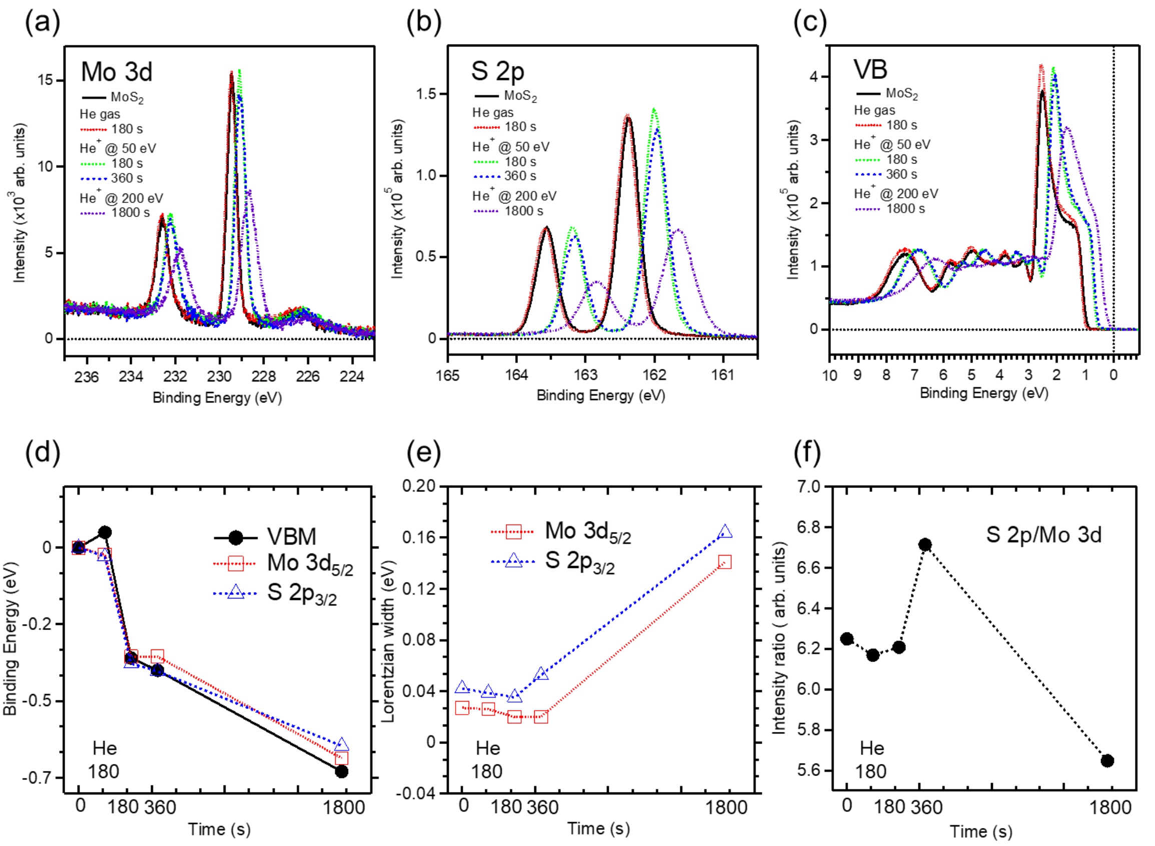

Figure S1. (a)-(c) Mo 3d, S 2p, and valence-band photoemission spectra were taken at the photon energies of $h\nu$ = 300 eV, 222 eV, and 56 eV, respectively. The Helium gas exposure of 180 Langmuir (L) (1 L = 10^{-6} Torr·s) induced negligible changes in all photoemission spectra of the freshly cleaved MoS_2 surface. However, the He^+ ion irradiation with increasing the ion energy and irradiation time moved all photoemission spectra toward the lower binding energy side with decreasing intensity and broadening linewidth. (d) The binding energy shifts of the prominent Mo $3d_{5/2}$ and S $2p_{3/2}$ peaks and the VBM. The binding energies of the in-situ cleaved MoS_2 surface are 229.47 eV, 162.38 eV, and 0.98 eV, respectively. (e) Comparison of Lorentzian linewidth of Mo 3d and S 2p core-level spectra. (f) The intensity ratio of S 2p to Mo 3d core-level spectra as a function of the irradiation time and energy of the He-ion beam.

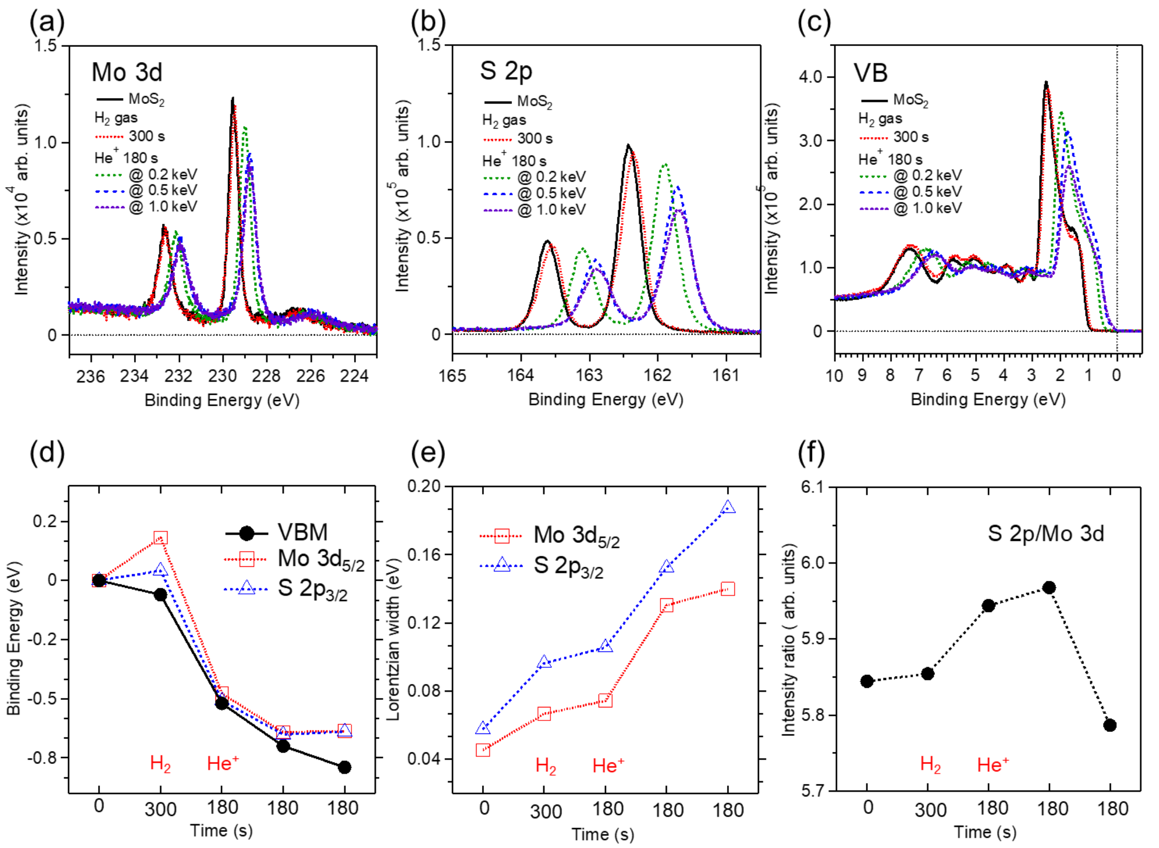

Figure S2. (a)-(c) Mo 3d, S 2p, and valence-band photoemission spectra were taken at the photon energies of *hv* = 300 eV, 222 eV, and 56 eV, respectively. The Hydrogen gas exposure of 300 L induced negligible changes in all photoemission spectra of the freshly cleaved MoS_2 surface. It means less production of mono-sulfur vacancy defects, which are known to very sensitive to the hydrogen gas [44], the current in-situ mechanical cleaving method. On the other hand, the He^+ ion irradiation with increasing the ion energy at the fixed irradiation time moved all photoemission spectra toward the lower binding energy side with decreasing intensity and broadening linewidth. (d) The binding energy shifts of the prominent Mo $3d_{5/2}$ and S $2p_{3/2}$ peaks and the VBM. The binding energies of the in situ-cleaved MoS_2 surface are 229.53 eV, 162.42 eV, and 1.03 eV, respectively. (e) Comparison of Lorentzian linewidth of Mo 3d and S 2p core-level spectra. (f) The intensity ratio of S 2p to Mo 3d core-level spectra as a function of the irradiation time and energy of the He-ion beam.

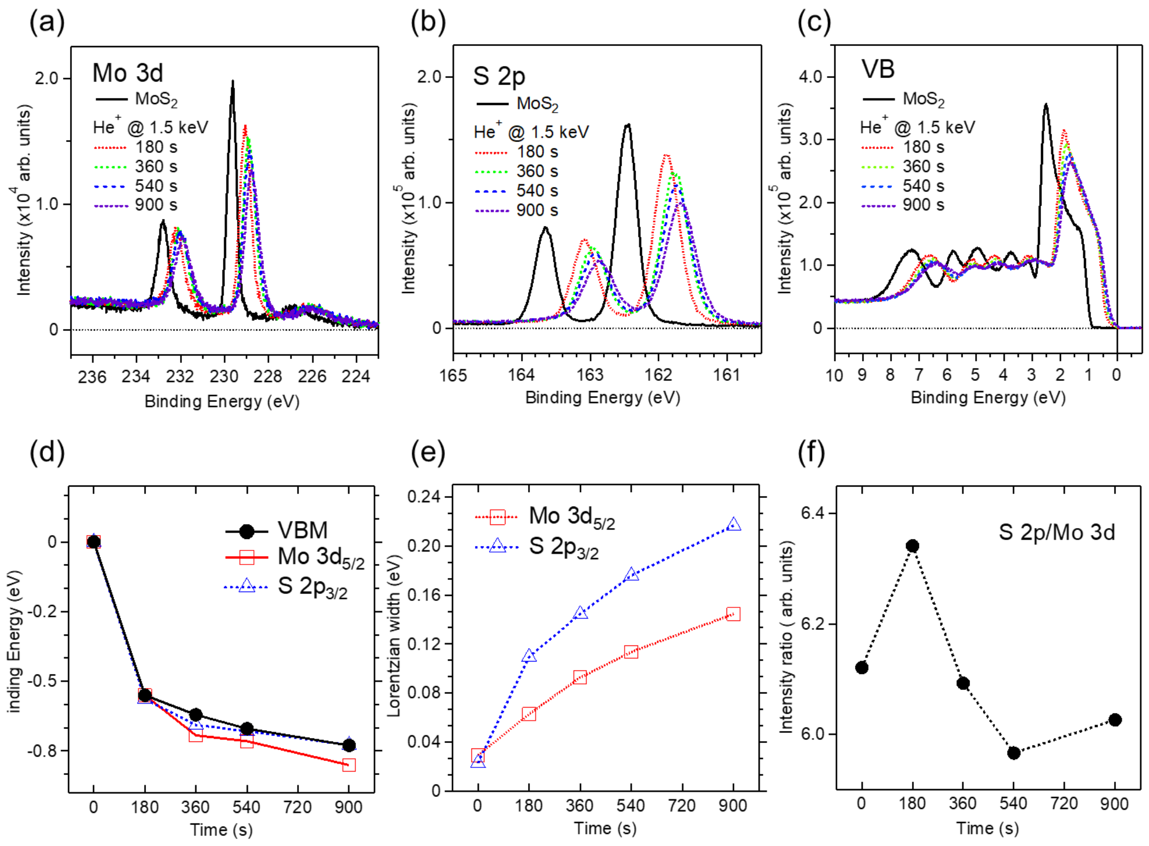

Figure S3. (a)-(c) Mo 3d, S 2p, and valence-band photoemission spectra were measured at the photon energies of $h\nu$ = 300 eV, 222 eV, and 56 eV, respectively. The He^+ ion irradiation with increasing the irradiation time at the fixed ion energy of 1.5 keV moved all photoemission spectra of the freshly cleaved MoS_2 surface toward the lower binding energy side with decreasing intensity and broadening linewidth. (d) The binding energy shifts of the prominent Mo $3d_{5/2}$ and S $2p_{3/2}$ peaks and the VBM. The binding energies of the in situ-cleaved MoS_2 surface are 229.65 eV, 162.47 eV, and 0.94 eV, respectively. (e) Comparison of Lorentzian linewidth of Mo 3d and S 2p core-level spectra. (f) The intensity ratio of S 2p to Mo 3d core-level spectra as a function of the irradiation time of the He-ion beam.

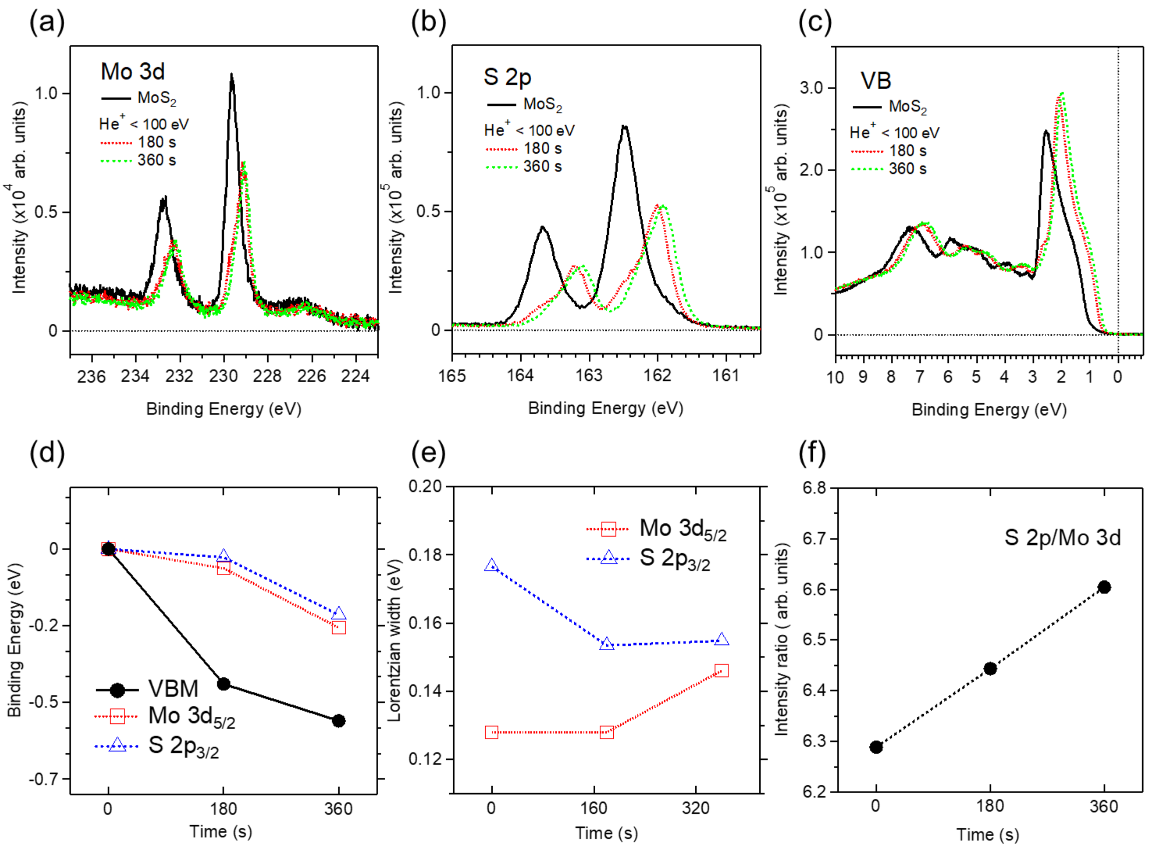

Figure S4. (a)-(c) Mo 3d, S 2p, and valence-band photoemission spectra were measured at the photon energies of *hν* = 300 eV, 222 eV, and 56 eV, respectively. The He^+ ion irradiation with increasing the irradiation time at the fixed ion energy below 100 eV shifts all photoemission spectra of the freshly cleaved MoS_2 surface toward the lower binding energy side with decreasing intensity and broadening linewidth. However, the high-energy (low-kinetic energy) components of all spectra remained. (d) The binding energy shifts of the prominent Mo $3d_{5/2}$ and S $2p_{3/2}$ peaks and the VBM. The binding energies of the in situ-cleaved MoS_2 surface are 229.64 eV, 162.49 eV, and 1.03 eV, respectively. (e) Comparison of Lorentzian linewidth of Mo 3d and S 2p core-level spectra. (f) The intensity ratio of S 2p to Mo 3d core-level spectra as a function of the irradiation time of the He-ion beam.

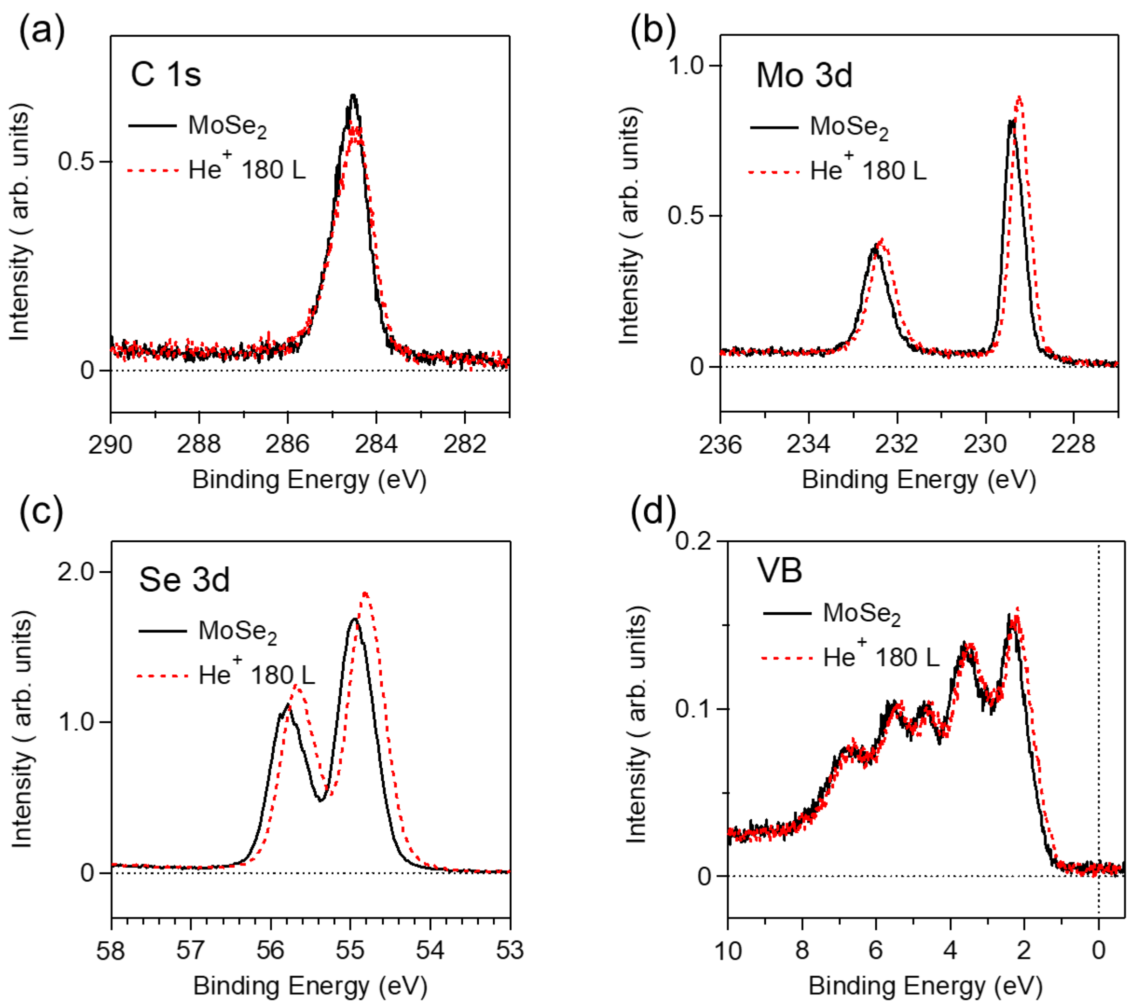

Figure S5. (a)-(d) Comparison of the C 1s, Mo 3d and Se 3d core-level spectra, and valence band spectra, taken at the photon energy of $h\nu$ = 360 eV. The low-energy (100 eV) He^+ ion irradiation moved $MoSe_2$-related photoemission spectra of the freshly cleaved surface toward the lower binding energy side, increasing intensities and broadening linewidths. By contrast, the adventitious C 1s core-level spectrum shifted toward the higher binding energy side, decreasing the intensity by forming asymmetric lineshapes due to the sputtering of the He^+ ion irradiation.

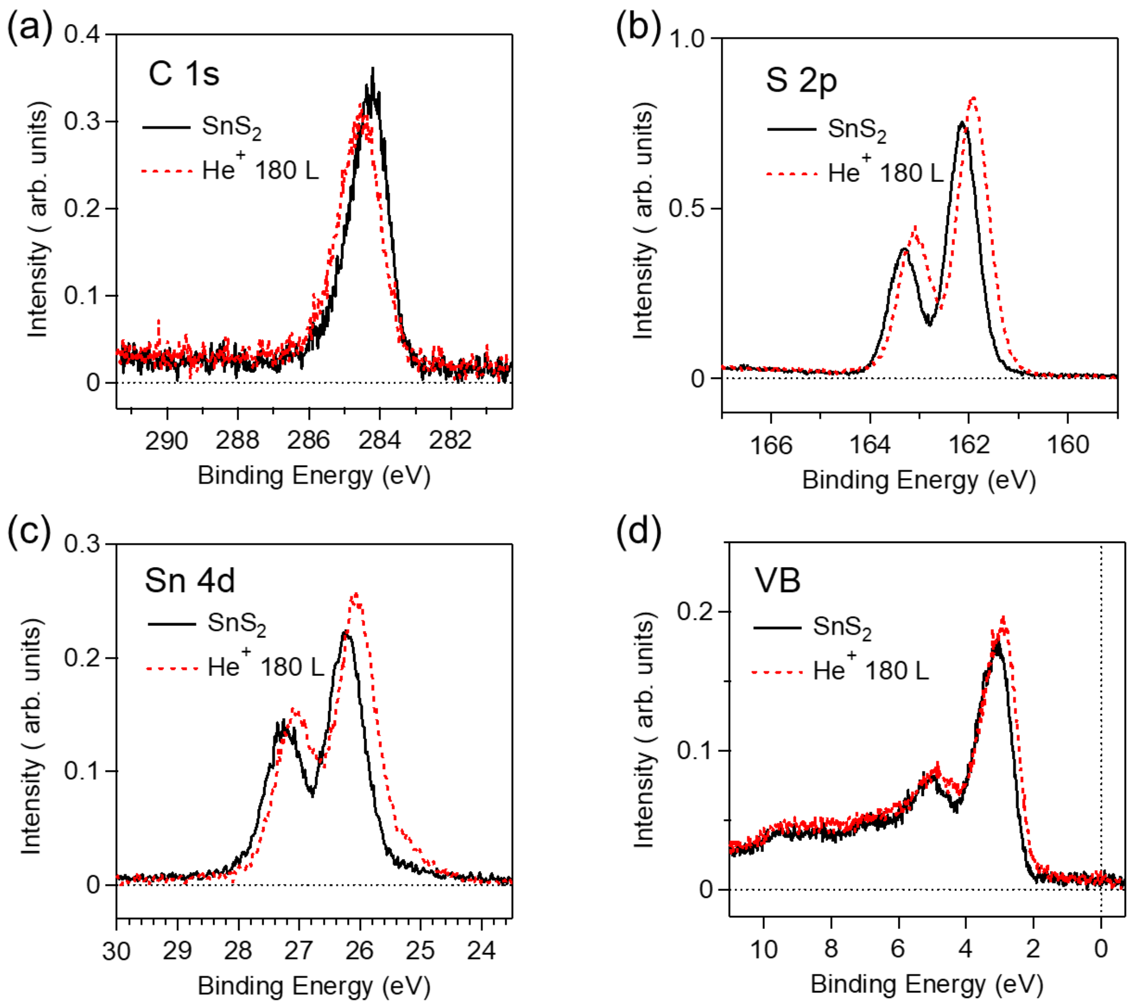

Figure S6. (a)-(d) Comparison of the C 1s, S 2p and Sn 4d core-level spectra, and valence band spectra, taken at the photon energy of $h\nu = 360$ eV. The low-energy (100 eV) He^+ ion irradiation moved SnS_2-related photoemission spectra of the freshly cleaved surface toward the lower binding energy side, increasing intensities and broadening linewidths. By contrast, the adventitious C 1s core-level spectrum shifted toward the higher binding energy side, decreasing the intensity by forming asymmetric lineshapes due to the sputtering of the He^+ ion irradiation.

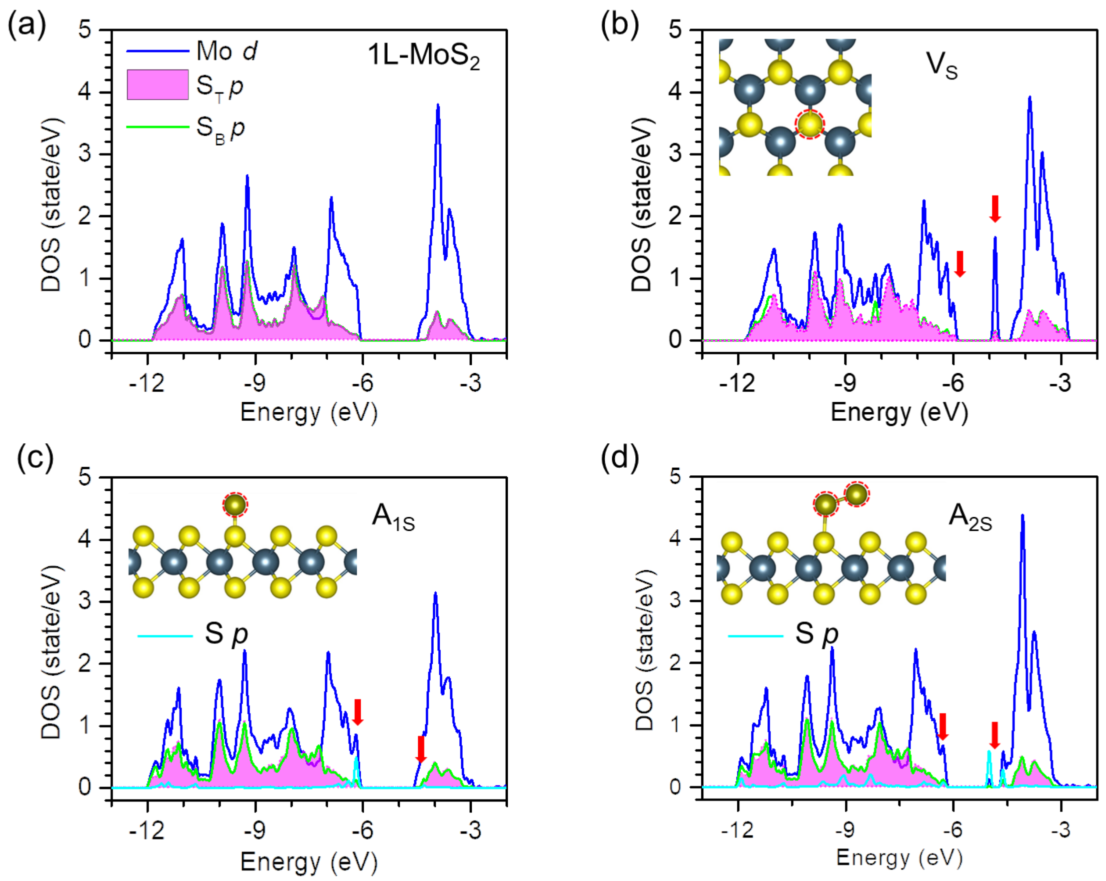

Figure S7. The calculated density of states (DOSs) of MoS_2 monolayer for (a) defect-free, (a) one S vacancy (V_{1S}), (c) one S adsorption (A_{1S}), and (d) two S adsorption (A_{2S}). The energy scale is aligned for the vacuum level.

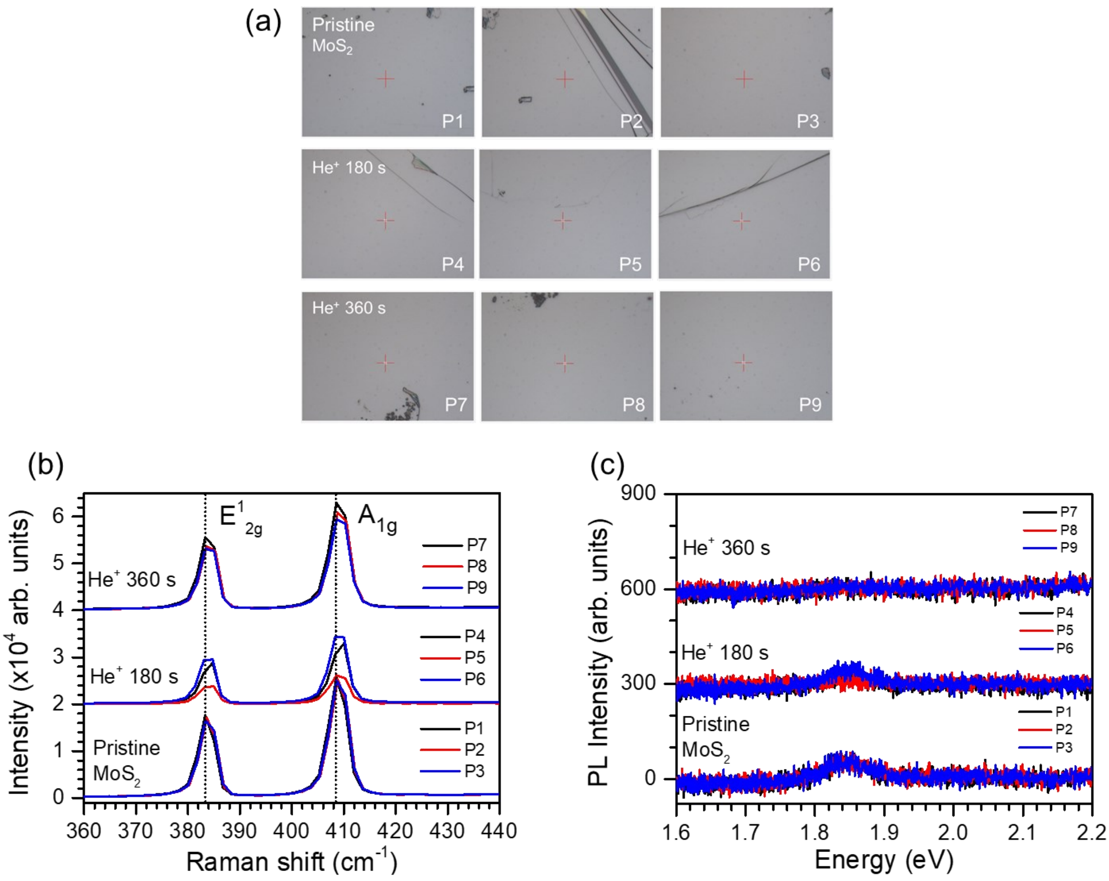

Figure S8. Comparison of (a) optical microscope images, (b) Raman, and (c) PL spectra. The bulk 2H-MoS_2 single crystals were mechanically exfoliated in ambient air. Some samples were irradiated by He^+ ion with an increasing irradiation time at the fixed He^+ ion energy of 100 eV.

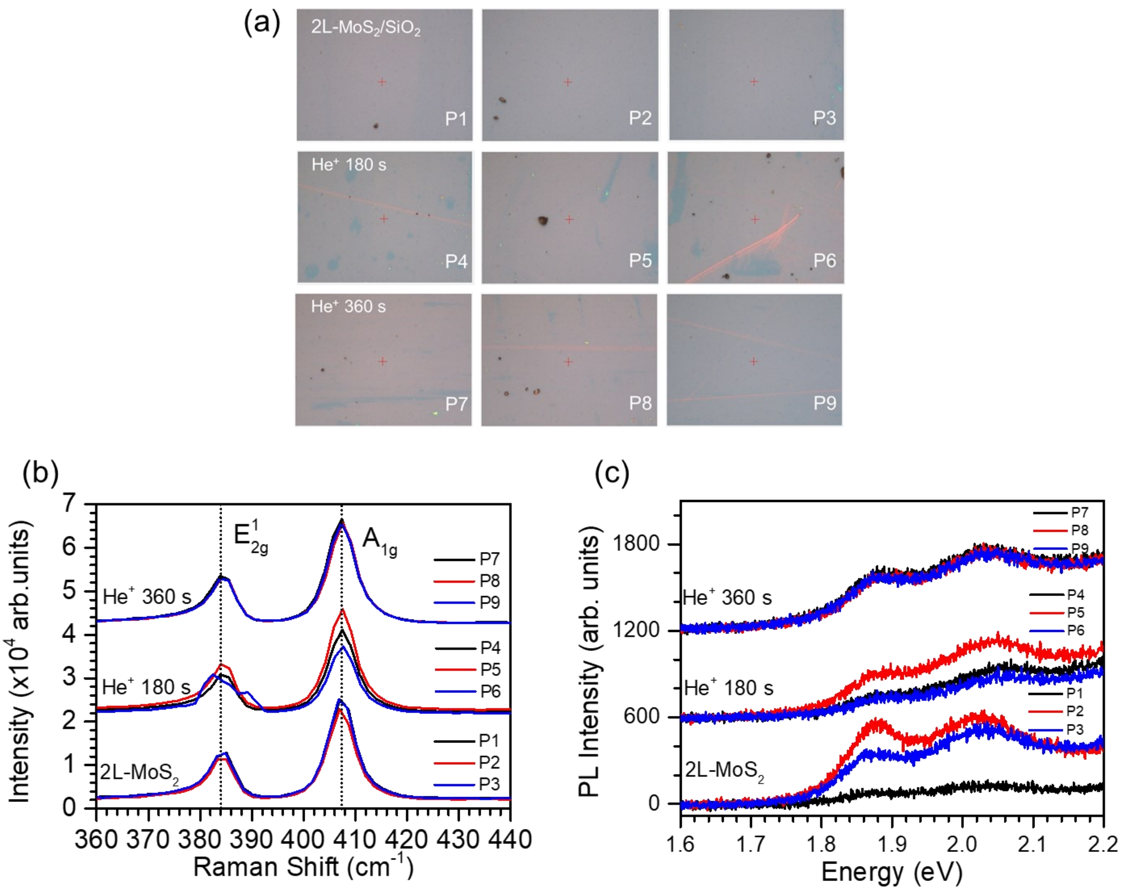

Figure S9. Comparison of (a) optical microscope images, (b) Raman, and (c) PL spectra. The CVD-grown 2L-MoS_2 samples on the SiO_2/Si substrates were exposed to ambient air. Some samples were irradiated by He^+ ion with an increasing irradiation time at the fixed He^+ ion energy of 100 eV.

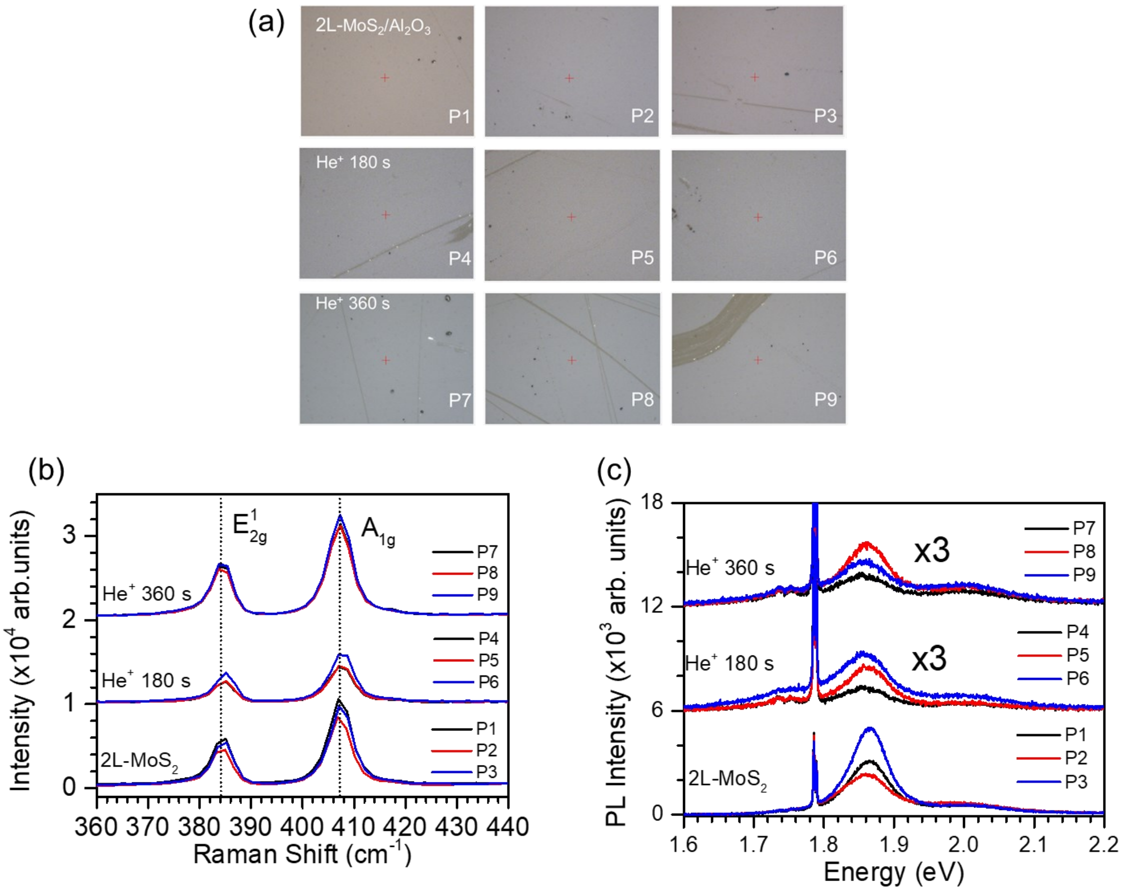

Figure S10. Comparison of (a) optical microscope images, (b) Raman, and (c) PL spectra. The CVD-grown 2L-MoS_2 samples on the Al_2O_3 substrates were exposed to ambient air. Some samples were irradiated by He^+ ion with an increasing irradiation time at the fixed He^+ ion energy of 100 eV.

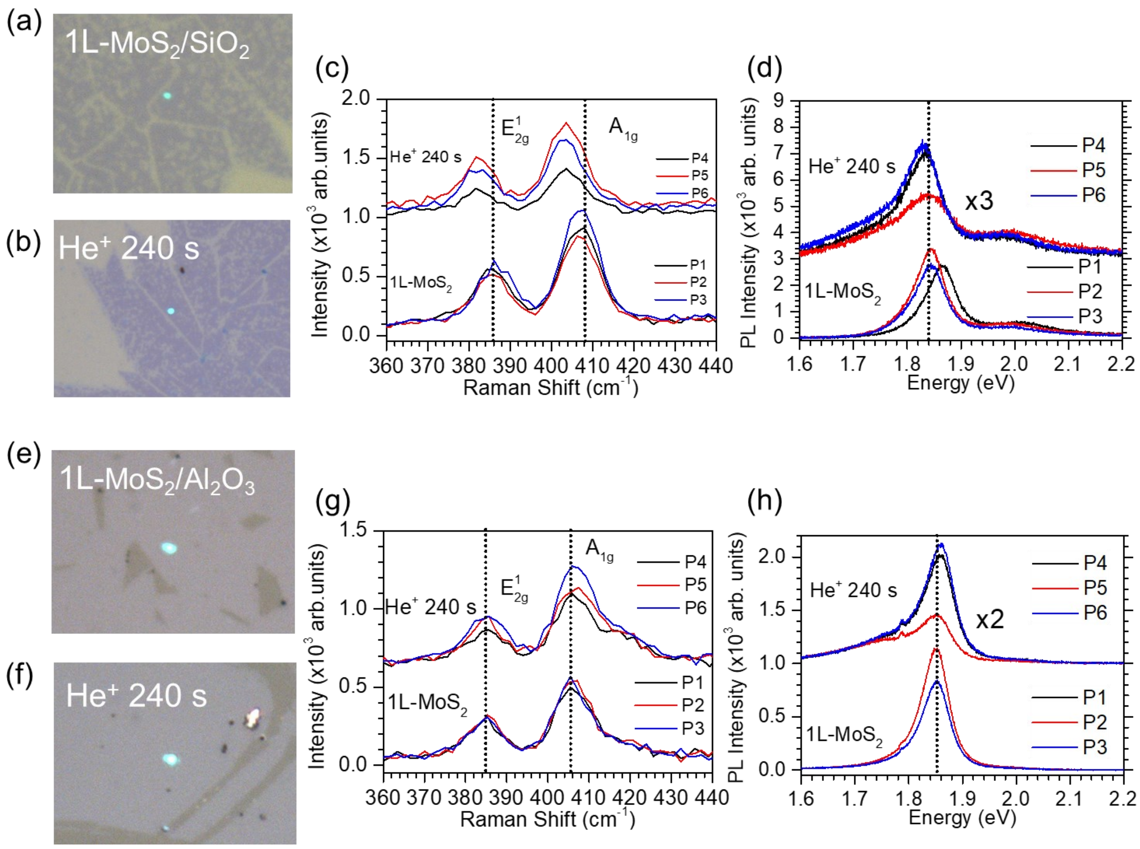

Figure S11. Comparison of (a,b,e,f) optical microscope images, (c,g) Raman, and (d,h) PL spectra of the CVD-grown 1L-MoS_2 on the SiO_2/Si (a-d) and Al_2O_3 (e-h) substrates. All samples were exposed to ambient air. Some samples were irradiated by He^+ ion for the 240 s at the fixed He^+ ion energy of 100 eV.

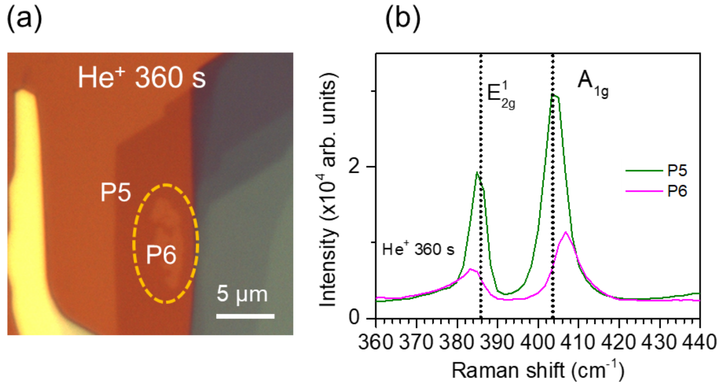

Figure S12. (a) Optical microscope images of the mechanically exfoliated 1L-MoS_2 on the SiO_2/Si substrate after the He^+ ion irradiation. (b), (c) Comparison of Raman and PL spectra obtained at other positions of Fig. 5(d).

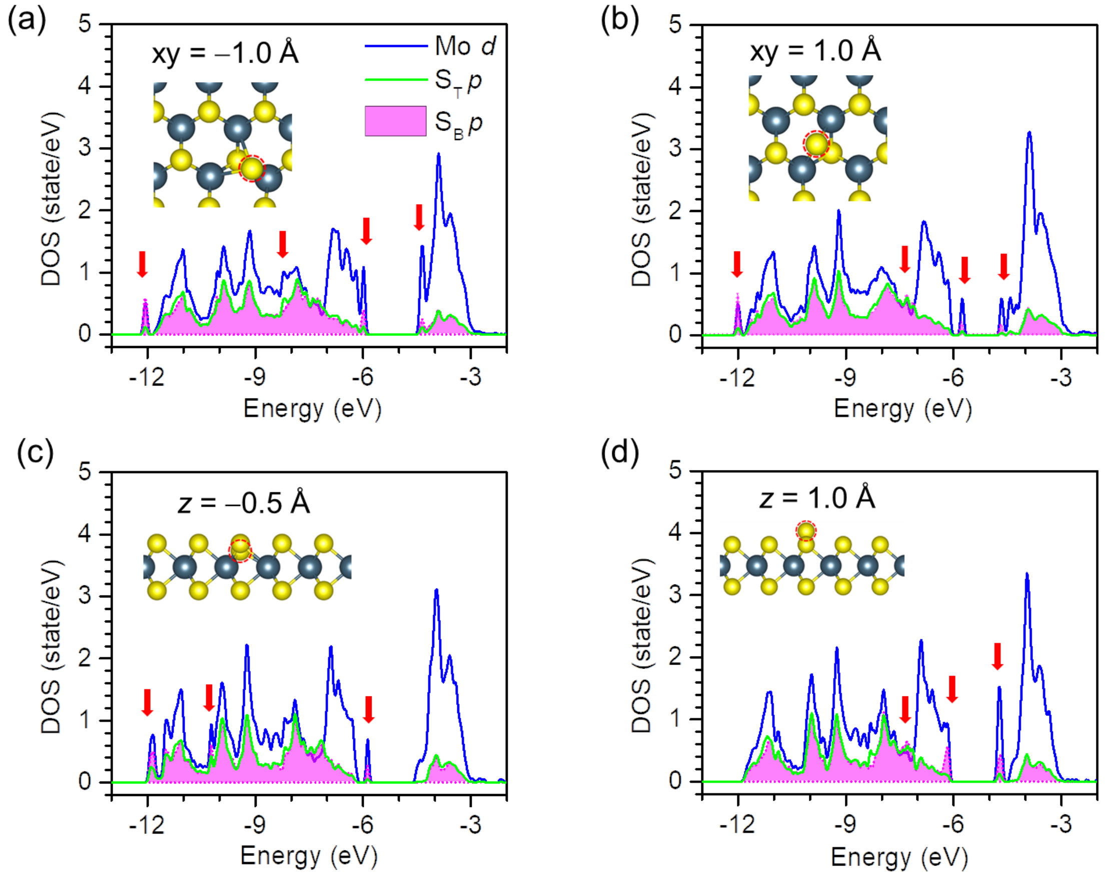

Figure S13. The calculated DOS of MoS_2 monolayer for (a) one S vacancy (V_{1S}), (b) the horizontal movement of an S atom, and (c),(d) the vertical movement of an S atom. The energy scale is aligned for the vacuum level.

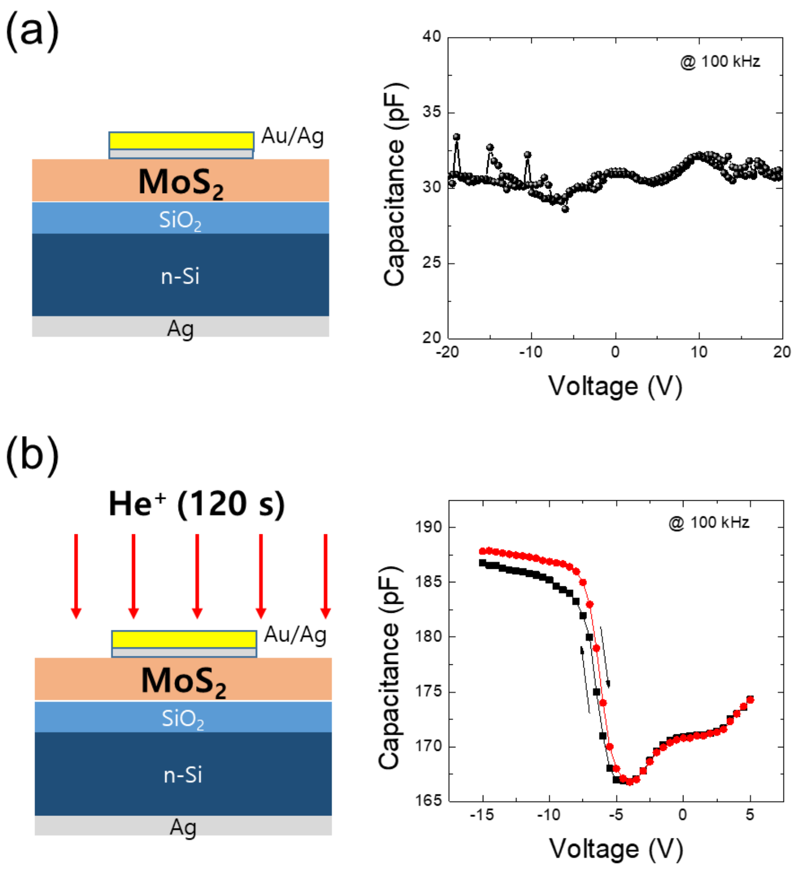

Figure S14. Schematic cross-sections of thicker MoS_2 MOS capacitor and capacitance-voltage curves measured at 100 kHz before (a) and after (b) He^+ ion irradiation for 120 s @ 100 eV ion energy.

Figure S14 shows no signal of the capacitance-voltage measurements of metal-oxide-semiconductor devices (MOS), i.e., thick MoS_2 on SiO/n-type Si substrate with the contacts of Ag(20 nm)/Au(100 nm) metals. It is essential to note that the structure differs from the conventional MOS capacitor structure [R1, R2]. However, relatively significant C-V characteristics were observed after the He^+ ion irradiation for 120 s. In contrast to the n-type MoS_2 [R1, R2], the accumulation region occurs at negative voltages, indicative of p-type MoS_2 [R3]. More examination with thin MoS_2 flakes could make it an ideal p-type 2D semiconductor for future electronic and optoelectronic applications.

References

R1. Das, S., Kumar, C., Kumar, R., Srivastava, A. & Jit, S. Two-Dimensional MoS_2-Based Photosensitive Al/MoS_2/SiO_2/Si/Ag MOS Capacitor. *IEEE Photonic. Tech. Lett*. **32**, 67-70 (2020).

R2. Yang, H. I. & Choi, W. Capacitance-Voltage Measurements of Monolayer MoS_2 Metal-Oxide-Semiconductor Capacitors. *Microelectron. Eng*. **238**, 111507 (2021).

R3. Xia, P. K. et al. Impact and Origin of Interface States in MOS Capacitor with Monolayer MoS_2 and HfO_2 High-k Dielectric. *Sci Rep*. **7**, 40669 (2017).
